# Tendon-motion tracking in an ultrasound image sequence using optical-flow-based block matching

**DOI:** 10.1186/s12938-017-0335-x

**Published:** 2017-04-20

**Authors:** Bo-I Chuang, Jian-Han Hsu, Li-Chieh Kuo, I-Ming Jou, Fong-Chin Su, Yung-Nien Sun

**Affiliations:** 1 Department of Computer Science and Information Engineering, 1 University Road, Tainan, 701 Taiwan; 2 Department of Occupational Therapy, 1 University Road, Tainan, 701 Taiwan; 3 Department of Orthopedics, E-Da Hospital, I-Shou University, 1 E-Da Road, Jiao-Shu Village, Yan-Chao District, Kaohsiung City, 82445 Taiwan; 4 Department of Biomedical Engineering, National Cheng Kung University, 1 University Road, Tainan, 701 Taiwan

**Keywords:** Tendon tracking, Ultrasound, Optical flow, Block matching

## Abstract

**Background:**

Tendon motion, which is commonly observed using ultrasound imaging, is one of the most important features used in tendinopathy diagnosis. However, speckle noise and out-of-plane issues make the tracking process difficult. Manual tracking is usually time consuming and often yields inconsistent results between users.

**Methods:**

To automatically track tendon motion in ultrasound images, we developed a new method that combines the advantages of optical flow and multi-kernel block matching. For every pair of adjacent image frames, the optical flow is computed and used to estimate the accumulated displacement. The proposed method selects the frame interval adaptively based on this displacement. Multi-kernel block matching is then computed on the two selected frames, and, to reduce tracking errors, the detailed displacements of the frames in between are interpolated based on the optical flow results.

**Results:**

In the experiments, cadaver data were used to evaluate the tracking results. The mean absolute error was less than 0.05 mm. The proposed method also tracked the motion of tendons in vivo, which provides useful information for clinical diagnosis.

**Conclusion:**

The proposed method provides a new index for adaptively determining the frame interval. Compared with other methods, the proposed method yields tracking results that are significantly more accurate.

**Electronic supplementary material:**

The online version of this article (doi:10.1186/s12938-017-0335-x) contains supplementary material, which is available to authorized users.

## Background

A tendon is a band of fibrous tissue that connects muscle to bone. Muscle contraction pulls the tendon, which causes the limbs to move; thus, tendon motion is important for evaluating the status of limb and joint functions. Tendinopathy of the finger, such as trigger finger (a.k.a. stenosing tenosynovitis), has become a frequent occupational disease in recent decades. A patient with trigger finger will need surgical treatment if the symptom is at a serious stage. The first annular pulley (A1 pulley) will be cut off to increase the sliding space of the tendon. The percutaneous release technique developed by Lorthioir [1] uses a specially designed knife to divide the pulley. Jou et al. [2] also proposed a new ultrasound-assisted minimally invasive surgical technique to increase the safety of percutaneous release. In these surgical techniques, ultrasound imaging is used to observe the tendon position and appearance. Some studies report that tendinopathy changes tendon behavior. Klauser et al. [3] found a significant difference in tendon stiffness between patients with diseased Achilles tendons and healthy controls. Sahu et al. [4] also found that tendinopathy limits the motion of tendons.

**Fig. 1:**
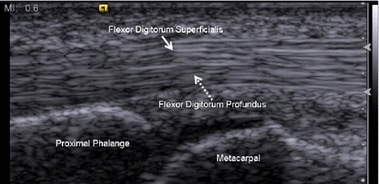
Finger ultrasound image at the A1 pulley

**Fig. 2:**
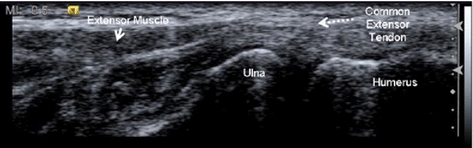
Elbow ultrasound image

Because ultrasound images are normally used to observe the characteristics and motion of different tissue types [5–7], images of two different upper-limb tendons, the flexor tendon in the finger and a common extensor tendon in the elbow, for tracking in this study. Tendinopathy of the flexor tendon will cause trigger finger, and in a common extensor tendon will cause tennis elbow. In the finger, the flexor tendon includes the flexor digitorum superficialis (FDS) (Fig. 1, solid arrow) and the flexor digitorum profundus (FDP) (Fig. 1, dashed arrow) tendons. The FDS and FDP have different motions at different angles of finger flexion and extension; thus, these two tendons cannot be tracked as one moving target. In the elbow, the common extensor tendon is a short connector between bone and muscle (Fig. 2, dashed arrow). The tendon will glide when the connected extensor muscle contracts. Although the tendon motion can be seen, speckle noise makes tracking the tendon in an ultrasound image sequence is difficult. Furthermore, if the direction of the acquisition probe does not parallel to the direction of the tendon motion, the ultrasound image sequence will be out-of-plane. We developed a tracking method that can track the tendon motion in the ultrasound video with speckle noise. The proposed method cannot track the tendon when the adjacent image frames are completely out-of-plane. Instead, if we place the probe parallel to the tendon motion direction during image acquisition, the proposed method can perform well by tracking the target using the residual information between frames.

In the past decade, optical flow and block matching have been the two most frequently used methods to track tissue using ultrasound [8–17]. Zahnd et al. [8] and Lai et al. [9] proposed the Kalman-based block matching method for carotid and tendon motion. Ayvali and Desai [10] and Tenbrinck et al. [11] applied the optical flow to track the motion of needle head and left ventricle. Furthermore, Barbosa et al. [12] developed a tracking method that combines optical flow and block matching for left ventricle motion in 4-D ultrasound sequences. Korstanje et al. [13] developed a multi-kernel ultrasound speckle tracking method to quantify tendon displacement and reduce the tracking error.

Although block matching and the optical flow method were used to track a moving target in ultrasound images in the above referenced studies, there are still several challenges that need to be resolved. First, the tracking error in the block matching method will increase over time [13]. In the block matching method, the sub-pixel displacement between frames usually generates a small amount of template error during template updating. This error will accumulate over time and will make the tracking results dissimilar to the initial tracking template. Thus, a template updating procedure should be used for appropriate frames to lower the tracking error. Second, the displacement cannot be too large when using the optical flow method [18]. Because of the intensity consistency constraint, the optical flow method will underestimate the displacement if the motion is too large. Finally, the tracking frame intervals in the block matching process can also affect the tracking results significantly and must be set. Dilley et al. [19] reported that different frame intervals affected the cross-correlation tracking method. In their cases, increasing the frame interval improved the accuracy of the tracking results for slower velocity data. Nevertheless, they did not provide a mechanism to automatically adjust the frame interval during image tracking. Although many methods use adjacent frames or constant frame intervals in block matching, an adaptive frame interval based on the properties of a tracked image sequence will make speckle tracking more flexible and accurate.

In this research, we propose an optical-flow-trend-based multi-kernel block matching (OFTB-MKBM) method that combines block matching with optical flow methods for automatic speckle tracking. The OFTB-MKBM is intended to provide a new index for adaptively determining the frame interval. Optical flow and MKBM methods are used to compare the tracking accuracy with the OFTB-MKBM, and adaptive MKBM using linear interpolation rather than the optical flow trend is evaluated to illustrate the effectiveness of this new method.

## Methods

### Optical flow method

The optical flow method is a classic tracking method [20]. Its primary assumption is an intensity consistency constraint that can be written:

1 $$I\left( {x,y,t} \right) = I\left( {x + \delta x,y + \delta y,t + \delta t} \right),$$

where *I*(*x*,*y*,*t*) means the intensity value of position (*x, y*) in the *t*th frame, and δ*x* and *δy* are the displacement differences after the time interval *δt*. Using the Taylor series, $I\left( {x + \delta x,y + \delta y,t + \delta t} \right)$ can be written:

2 $$I\left( {x + \delta x,y + \delta y,t + \delta t} \right) = I\left( {x,y,t} \right) + \frac{\delta I}{\delta x}\Delta x + \frac{\delta I}{\delta y}\Delta y + \frac{\delta I}{\delta t}\Delta t + H.O.T.$$

where *H.O.T.* means “higher order terms”.

Combining and reducing Eqs. (1) and (2), can be rewritten:

3 $$I_{x} V_{x} + I_{y} V_{y} = - I_{t} ,$$

where *V*_*x*_ and *V*_*y*_ are the *x* and *y* components of velocity (or displacement) at position (*x*,*y*) in the *t*th frame, and *I*_*x*_, *I*_*y*_, and *I*_*t*_ are the derivatives of the pixels at (*x*, *y*, and *t*) in the *x*, *y*, and *t* dimensions. Lucas and Kanade [21] presented a differential method for estimating optical flow. They assumed that velocity flows in a small region are similar. Thus, Eq. (3) can be resolved by rewriting it in matrix form with the pixels in a small region:

4 $$\left[ {\begin{array}{*{20}c} {I_{x} \left( {p_{0} } \right)} & {I_{y} \left( {p_{0} } \right)} \\ {I_{x} \left( {p_{1} } \right)} & {I_{y} \left( {p_{1} } \right)} \\ \vdots & \vdots \\ {I_{x} \left( {p_{n} } \right)} & {I_{y} \left( {p_{n} } \right)} \\ \end{array} } \right]\left[ {\begin{array}{*{20}c} {V_{x} } \\ {V_{y} } \\ \end{array} } \right] = - \left[ {\begin{array}{*{20}c} {I_{t} \left( {p_{1} } \right)} \\ {I_{t} \left( {p_{2} } \right)} \\ \vdots \\ {I_{t} \left( {p_{n} } \right)} \\ \end{array} } \right],$$

where *p*_*n*_ is the *n*th neighbor point in the computing region. *I*_*x*_, *I*_*y*_, and *I*_*t*_ are the derivatives of the pixels at (*x*, *y*, and *t*) in the *x*, *y*, and *t* dimensions. *V*_*x*_ and *V*_*y*_ are the *x* and *y* components of velocity at position (*x*,*y*).

### Multi-kernel block matching

Block matching is a detection and tracking method in image processing. It is used to compute the similarity between a reference block and a target block. However, block matching is sensitive to speckle noise. The speckle noise is the small scale brightness variations of speckle which affect the tracking results when the variations are significant.

Korstanje et al. [13] proposed an MKBM scheme to solve this problem. MKBM is a multi-kernel block matching method that separates the reference block into several sub-blocks. Each sub-block is initially examined using the block matching method to find the block that is the closest match. The matching results of all sub-blocks are then combined to obtain the overall matching result. By utilizing the multiple block matching, MKBM computes the normalized-cross-correlation (NCC) weighted average as the tracking result which is less affected by the speckle variations. However, MKBM still cannot perform well if the motion of tracking target is too small.

### Optical-flow-trend-based multi-kernel block matching

**Fig. 3:**
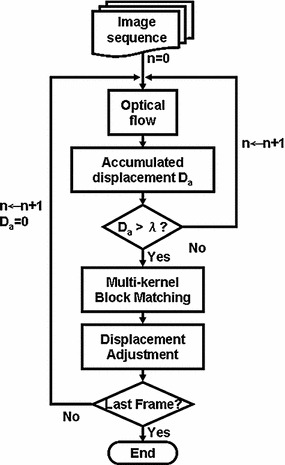
Flowchart of OFTB-MKBM

Both the optical flow and MKBM methods have advantages and disadvantages. The optical flow method can track and evaluate the target displacement when there is a small amount of motion, but it will fail if the target’s motion is too large. The MKBM method can track the target motion between large time intervals; however, the tracking cannot perform well if the motion of tracking target is too small. Thus, we propose a tracking structure that combines the advantages of both: an optical-flow-trend-based multi-kernel block matching method. The optical flow method is first used to compute the displacement in adjacent frames (Fig. 3). The MKBM is then used in the two selected frames: the starting frame and the frame with an accumulated displacement larger than a given constant (λ). The detailed displacements of MKBM between selected frames are finally adjusted based on the results using the optical flow method. The process is repeated until all of the input images are completed.

**Fig. 4:**
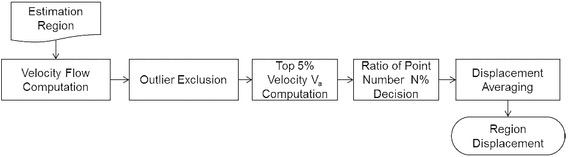
Flowchart of the optical flow method in our proposed procedure

In the optical flow method, the changes of region size used to compute the velocity flow will obtain different results. Since a tendon is non-rigid tissue, tendon deformation usually occurs with motion, and an estimated region that is too large will lead to the wrong result because the motions inside the region conflict with the assumption of the optical flow method because of deformation. However, if the region is too small, the tracking result will be severely affected by noise. A procedure to resolve these problems have been developed (Fig. 4).

**Fig. 5:**
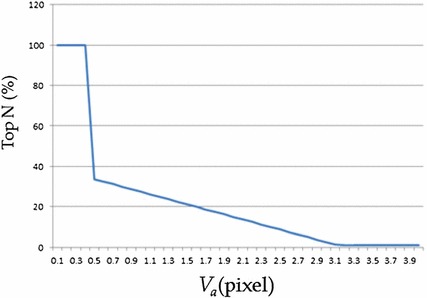
*V*_*a*_-N line chart

We computed several velocity flows inside the rectangular region to obtain the region displacement. The velocities of all the flow points inside the region were calculated using the optical flow method. Throughout the experiment, the window size for computing the optical flow of each flow point was 17 × 17 pixels. Inside a 101 × 41-pixel region, 43 × 13 flow points (with 2-pixel increments in both the *x* and *y* directions) were calculated and used to determine the region displacement. Because the major tendon motion is horizontal (*x* direction), only the horizontal motions of the region were used when determining the MKBM step size. To exclude outliers, the flow points were classified based on their motion direction in the horizontal axis by using the following equation:

5 $$Dir\left( p \right) = \left\{ {\begin{array}{ll} {{\text{left}},} & {{\text{if }}V\left( p \right) <0;} \\ {\text{right,}} & {{\text{if }}V\left( p \right)>0;} \\ {\text{ignored,}} & {otherwise,} \\ \end{array} } \right.$$

where *V*(p) is the horizontal displacement of *p*, the flow point. The points with major motion direction that contains the most flow points are retained and used to calculate the displacement of the region. However, not all of the retained flow points are precise in displacement; thus, only partially retained flow points should be used to calculate the displacement. To determine the region displacement, we conducted an experiment to find the statistical relationship between the actual region displacement and the top 5% displacement of the flow points. Three hundred adjacent frame pairs were used to determine this relationship. For each adjacent frame pair, the traditional optical flow method was used to calculate the flow points in the target region. The average top 5% displacement *V*_*a*_ inside the region calculated as an index. We manually tracked the tendon motion for each adjacent frame pair. For each target region, by referring to the obtained *V*_*a*_, we arrived at a ratio with the top N % average displacement inside the region was equal to the manually tracked displacement. From the experiment, the relationship between *V*_*a*_ and N was plotted as follows (Fig. 5).

In the implementation, we chose the number of flow points (N) conveniently based on *V*_*a*_. For example, if the average displacement of the top 5% flow points is 1.5 pixels (*V*_*a*_ = 1.5), the accurate displacement should be computed using the top 20% flow points (N = 20). The region displacement can then be obtained by averaging the displacements from the specific number of flow points.

**Fig. 6:**
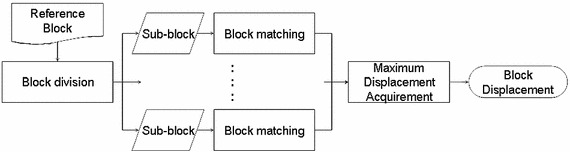
Flowchart of MKBM

The result of the optical flow method was used to compute the accumulated displacement (*D*_*a*_). If the magnitude of *D*_*a*_ was less than a predefined threshold value λ, the optical flow method was repeated with the subsequent frame. If *D*_*a*_ was larger than λ, the optical flow computing was then terminated and formed a flow period. Within the flow period, the MKBM method was then applied to the starting frame (*t*) and the end ending frame (*t* + *n*). In the MKBM procedure, a suitable algorithm for tendon tracking is proposed (Fig. 6).

As in the method described in Korstanje et al. [13], we first divided the reference block into four sub-blocks with ten overlapping pixels. Taking account of computational speed, rather than using normalized correlation coefficient, the sum of absolute differences (SAD) is used as the similarity measurement for each sub-block:

6 $$SAD = \frac{1}{MN}\sum\limits_{j = 1}^{N} {\sum\limits_{i = 1}^{M} {\left| {T_{i,j} - R_{i,j} } \right|} } ,$$

where M and N are the width and height of the sub-block, and *T*_*i,j*_ and *R*_*i,j*_ are the intensity values of pixels (*i*, *j*) at the target block and reference block, respectively. Because the soft tissue adjacent to the tendon might passively move with a smaller displacement, we computed the block displacement by choosing the maximal value of the four sub-blocks:

7 $${\text{D}}_{\text{t}} = {\text{Max(D}}_{\text{t,1}} ; {\text{ D}}_{\text{t,2}} ; {\text{ D}}_{\text{t,3}} ; {\text{ D}}_{\text{t,4}} ) ,$$

where *D*_*t,n*_ is the displacement of *n*th sub-block at the *t*th frame. Although the displacement between the starting and ending frames was obtained, the detailed displacements between the selected frames were unknown. Because the optical flow method can track the target with little underestimation for small motion displacement, the displacement of each frame between *t* and *t* + *n* can be interpolated using the results of the optical flow method and MKBM method:

8 $$d_{OFTB\_MKBM} (t + i) = d_{MKBM} (t + i) + (d_{OF} (t + i)-d_{OF} (t))\times\frac{{d_{MKBM} (t + n)-d_{MKBM} (t)}}{{d_{OF} (t + n)-d_{OF} (t)}},\quad 0 \le i \le n,$$

where *d*_*MKBM*_(t) and *d*_*OF*_(t) are the displacements at the *t*th frame computed using the MKBM and optical flow methods, respectively.

## Results and discussion

### Data acquisition

**Fig. 7:**
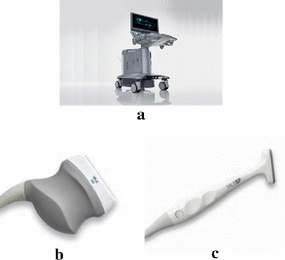
The ultrasound machine and the transducers. **a** ACUSON S2000 ultrasound system; **b** Siemens ACUSON S2000 18L6 HD ultrasound transducer; **c** Siemens ACUSON S2000 14L5 SP ultrasound transducer

The ultrasound image data (Additional files 1, 2) were acquired from National Cheng Kung University Hospital using the ACUSON S2000 Ultrasound System (Siemens Medical Solutions, Mountain View, CA, USA, Fig. 7a) with different settings for elbow and finger motions. Prior to image acquisition, all participants were informed about the aims and procedures of study, and signed consent forms approved by the Institutional Review Board of National Cheng Kung University Hospital (IRB number: B-ER-101-012). The image of acquired video is 1024 × 768 in size, and the frame rate is 30 fps. Since our algorithm is not dedicated to a single system, two different transducers were used due to the hardware constraint. In elbow data acquisition, the 18 MHz transducer (Fig. 7b) is used to acquire the ultrasound images with a pixel resolution of 0.075 mm/pixel. The subjects laid their right arm on the table with palm facing down with wrist extension pose. They were asked to virtually push the hard plate, which is fixed and unmovable, by extending and releasing their wrist. An ultrasound probe was placed above the lateral epicondyle parallel to the tendon direction. In finger cases, a 14 MHz transducer (Fig. 7c) was used to acquire the ultrasound images with a pixel resolution of 0.0265 mm/pixel. The subjects also laid their right arm on the table but with the palm facing up and the finger flexion pose. The subjects were asked to virtually push a hard plate by flexing their PIP or DIP joints. An ultrasound probe was placed above the A1 pulley region parallel to the tendon direction. An ultrasound expert blinded to the results of proposed method is asked to track both the finger and elbow tendon as the ground truths of in vivo cases (Additional files 3, 4).

**Fig. 8:**
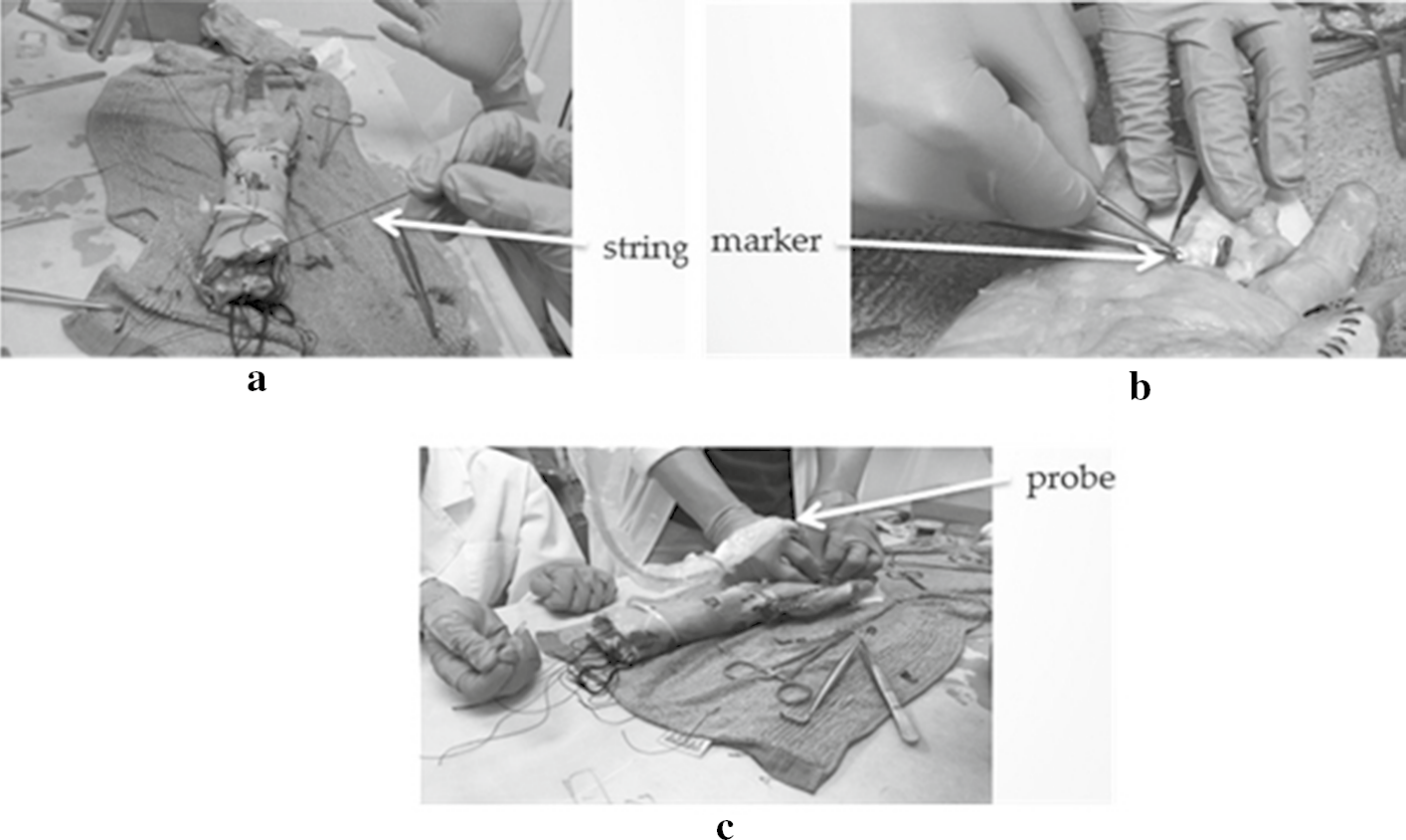
The cadaver’s hand used to acquire the ultrasound images. **a** The tendon tied with a string and passed through the entire forearm of the cadaver; **b** the marker inserted in the FDP tendon; **c** the acquisition probe placed above the A1 pulley position

**Fig. 9:**
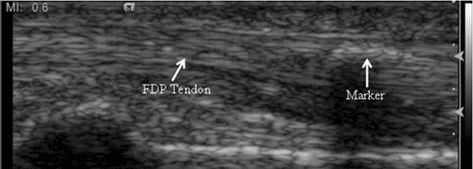
Ultrasound image of finger area in cadaver

In order to evaluate the accuracy of the proposed method, finger tendon images acquired from cadaver were also used (Additional file 5). To simulate the finger motion in the cadaver, we pressed the cadaver’s finger, which was then released by the weights hung on the tendon with a string at the elbow side, as shown in Fig. 8a. A tiny iron plate was inserted in FDS tendon close to the A1 pulley (Fig. 8b) as a marker. The ultrasound probe was placed above the region covering both the A1 pulley and the marker (Fig. 8c). The acquired cadaver ultrasound image is shown in Fig. 9.

### Determining window size

**Fig. 10:**
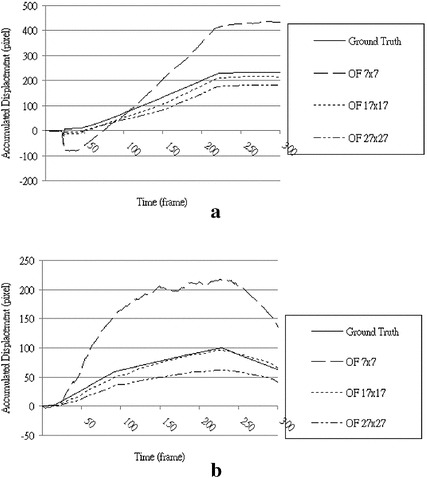
Two resulting displacements comparing the use of 3 different window sizes with the ground truths. OF 7 × 7, OF 17 × 17, and OF 27 × 27 represent window sizes 7 × 7, 17 × 17, and 27 × 27, respectively. **a** Case 1, **b** Case 2

In this experiment, different settings were used to evaluate the window sizes for the optical flow method and OFTB-MKBM in block matching. To obtain the optimal window size for the optical flow method, we used 43 × 13 flow points inside a 101 × 41-pixel region with three window sizes: 27 × 27, 17 × 17, and 7 × 7. Sixteen image sequences were used to test the performance of different window sizes. Figure 10 shows the two prominent cases using the optical flow method with three different window sizes compared with the ground truths. For most of the cases, the resulting displacements with a window size of 17 × 17 were closer to the ground truths. The resulting displacements with large window sizes of 27 × 27 were slower than the ground truths because of local artifacts and deformations. The resulting displacements with a small window size of 7 × 7 yielded huge errors because of ambiguous solutions and noise variations. We conclude that the optical flow method is reliable regardless of window size.

**Fig. 11:**
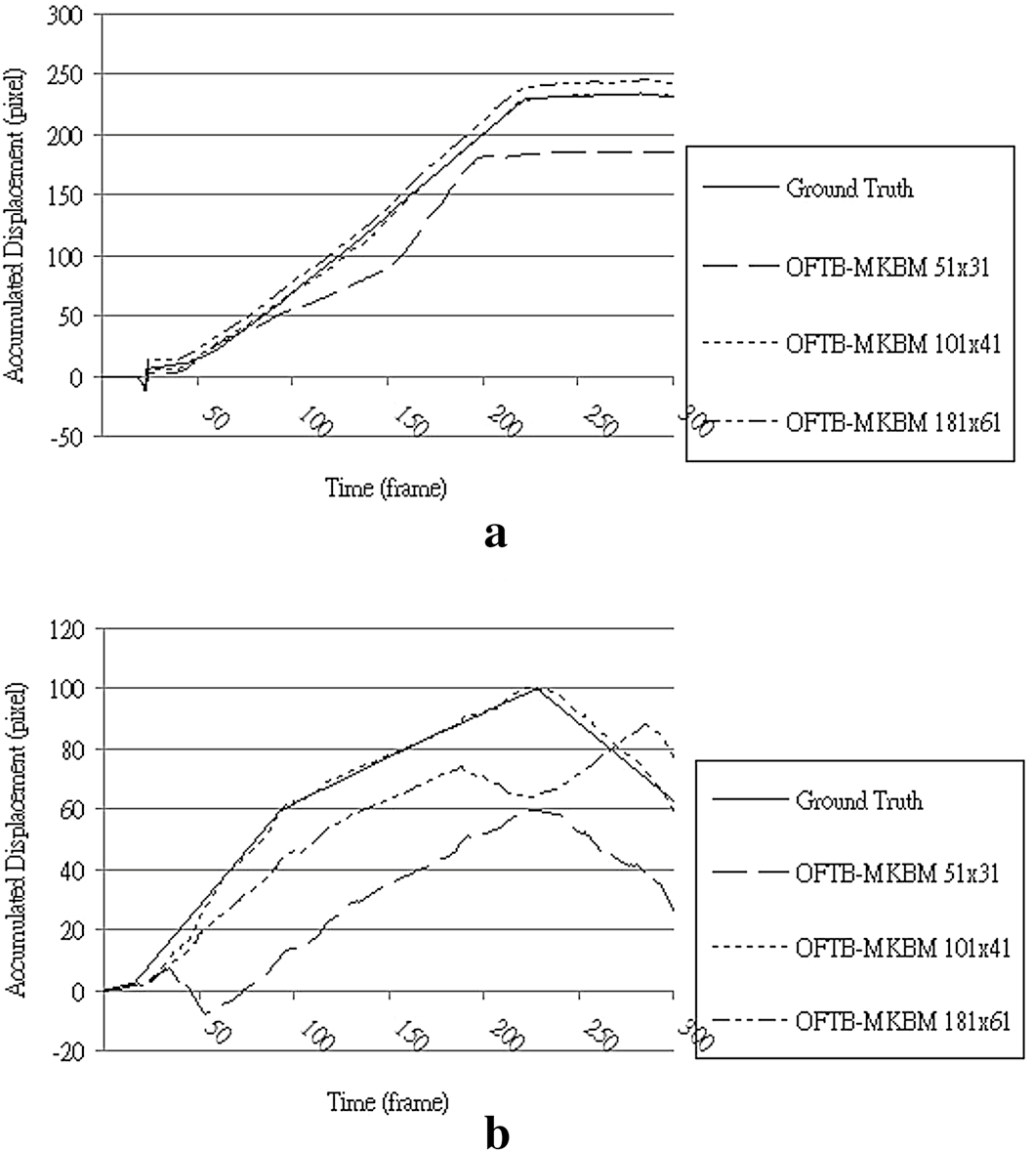
Two resulting displacements comparing the OFTB-MKBM with 3 different window sizes (181 × 61, 101 × 41, and 51 × 31) with the ground truths. **a** Case 1, **b** Case 2

To determine the optimal block size for OFTB-MKBM, three block sizes, 181 × 61, 101 × 41, and 51 × 31 in MKBM, were used and compared with the ground truth in sixteen image sequences to find the best setting. Each block was separated into four sub-blocks with 10 pixels overlapping in both dimensions. Two prominent tracking cases with these blocks are shown in Fig. 11. The resulting OFTB-MKBM displacements with a block size of 101 × 41 mostly match the ground truth in all cases. Cases with a small window size of 51 × 31 were typically less than the ground truth in both instances because of the influence of local noise. Case 1, with a large window size of 181 × 61, yielded a tracking result close to the ground truth, but in Case 2, the large window caused the wrong tracking direction in frames 180–290, which was confusing and led to a significant amount of error.

### Threshold of accumulated displacement

**Fig. 12:**
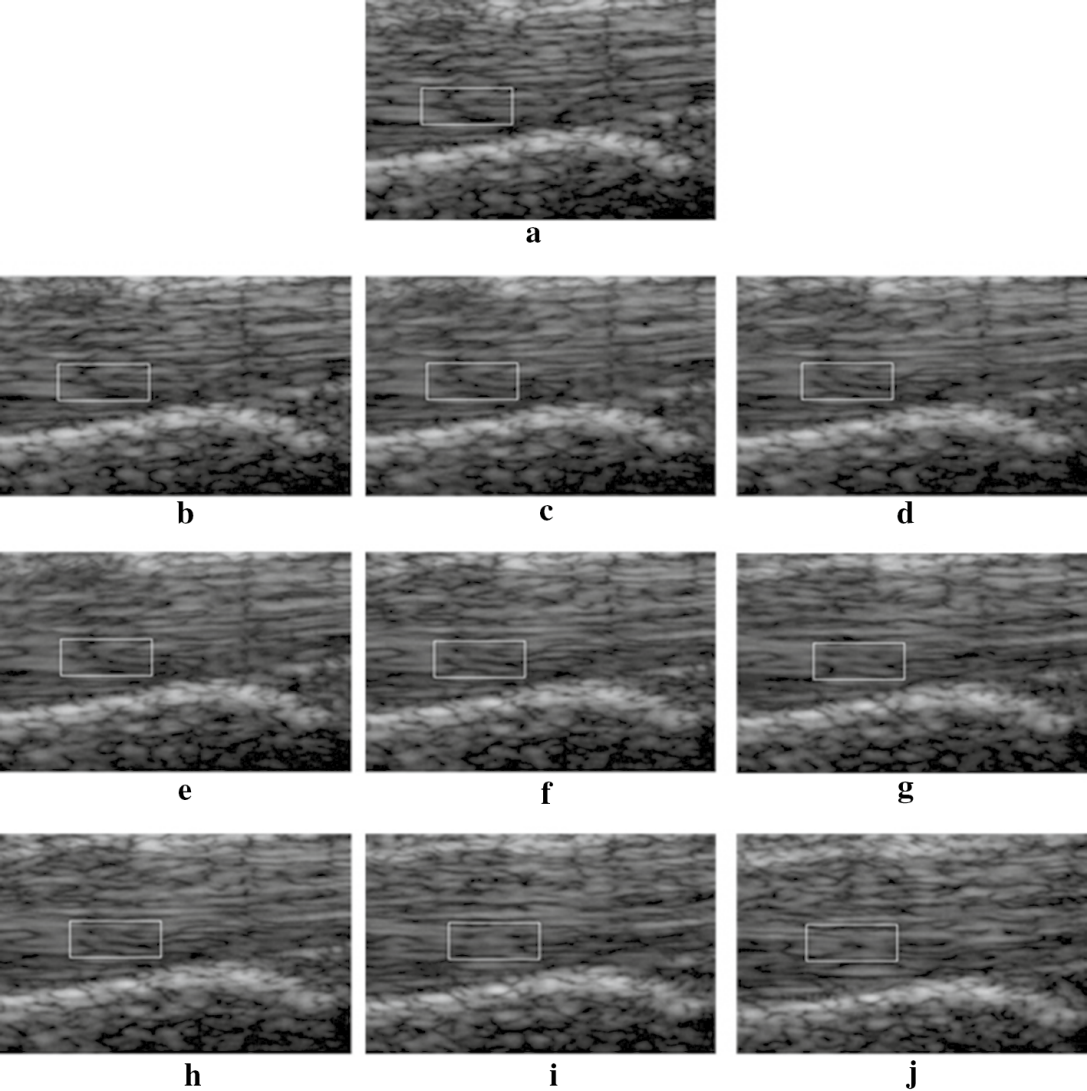
The tracking results of OFTB-MKBM with different λ values. **a** Initial reference template for frame 0; **b**–**d** with λ = 5 at frames 21, 25, and 30, respectively; **e**–**g** with λ = 10 at frames 24, 37, and 47, respectively; **h**–**j** with λ = 20 at frames 36, 62, and 96, respectively

In the proposed OFTB-MKBM, the adaptation of threshold λ, which determines when the MKBM should be used, is important. Higher λ values make the tracking faster; however, the tracking accuracy will decrease. Thus, we used the proposed OFTB-MKBM to evaluate tendon motion data with different λ settings for the sixteen image sequences. Some tracking results of selected frames are shown in Fig. 12. Figure 12a is the tracking region at the initial frame; (b–d) are the tracking results with λ values of 5; (e–g) are the tracking results with λ values of 10; and (h–j) are the tracking results with λ values of 20. The tracking results of λ = 5 and λ = 10 retained the texture of the tracking region. However, when λ = 20, the textures of each pair of adjacently selected frames the tracking regions became dissimilar. Although a larger λ value might reduce the computational cost of MKBM, we defined the threshold value λ as 10 pixels in the subsequent experiments for all the tendon motion image sequences. The selected λ value yielded satisfactory results in all acquired image sequences in our tendon motion experiments. If the method is used for cases with much faster motions, the λ value can be empirically adjusted to better fit the motions in these very different image sequences.

### Validating accuracy using standard ultrasound phantom

**Fig. 13:**
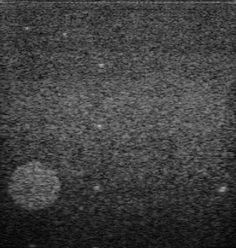
Phantom ultrasound image

**Fig. 14:**
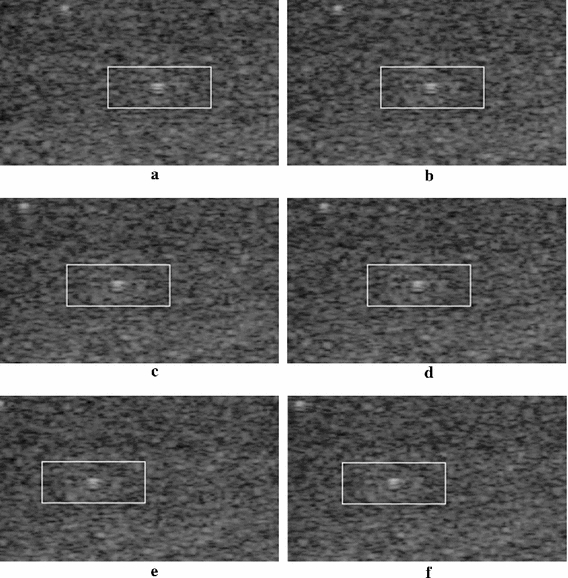
The tracking results of OFTB-MKBM in CIRS phantom. **a** Initial frame, **b** frame 48, **c** frame 83, **d** frame 118, **e** frame 149, **f** frame 182

In this experiment, the computerized imaging reference systems (CIRS) tissue-mimicking phantom (CIRS, Norfolk, VA) is used to quantize the tracking error of proposed method. We attached the ultrasound probe to a motor platform and recorded the ultrasound video of CIRS phantom while probe moving. Figure 13 is the acquired phantom ultrasound images. The moving speed of probe is 0.5 mm/s for 10 s, and the total displacement is 5 mm in 206 frames. Both the optical flow and the MKBM methods were compared with the proposed method. The total tracking displacement of optical flow, MKBM, and OFTB-MKBM are 3.77, 4.07 and 4.73 mm. The proposed OFTB-MKBM obtains the best tracking results than the other two methods. Comparing the results of proposed OFTB-MKBM and MKBM, the proposed method has smaller tracking error (0.27 mm) than MKBM (0.93 mm) since the matching frames of proposed method are adaptively selected based on the information of optical flow. The selected frames are first matched by MKBM and the motions of in-between adjacent frames are then adjusted by using the optical flow. The proposed method takes the advantage of both optical flow and MKBM, and thus the resulting tracking error is much smaller than the one of MKBM. Figure 14a–f are the tracking results. The textures inside the tracking region are similar in all the frames.

### Validating accuracy using cadaver data

**Fig. 15:**
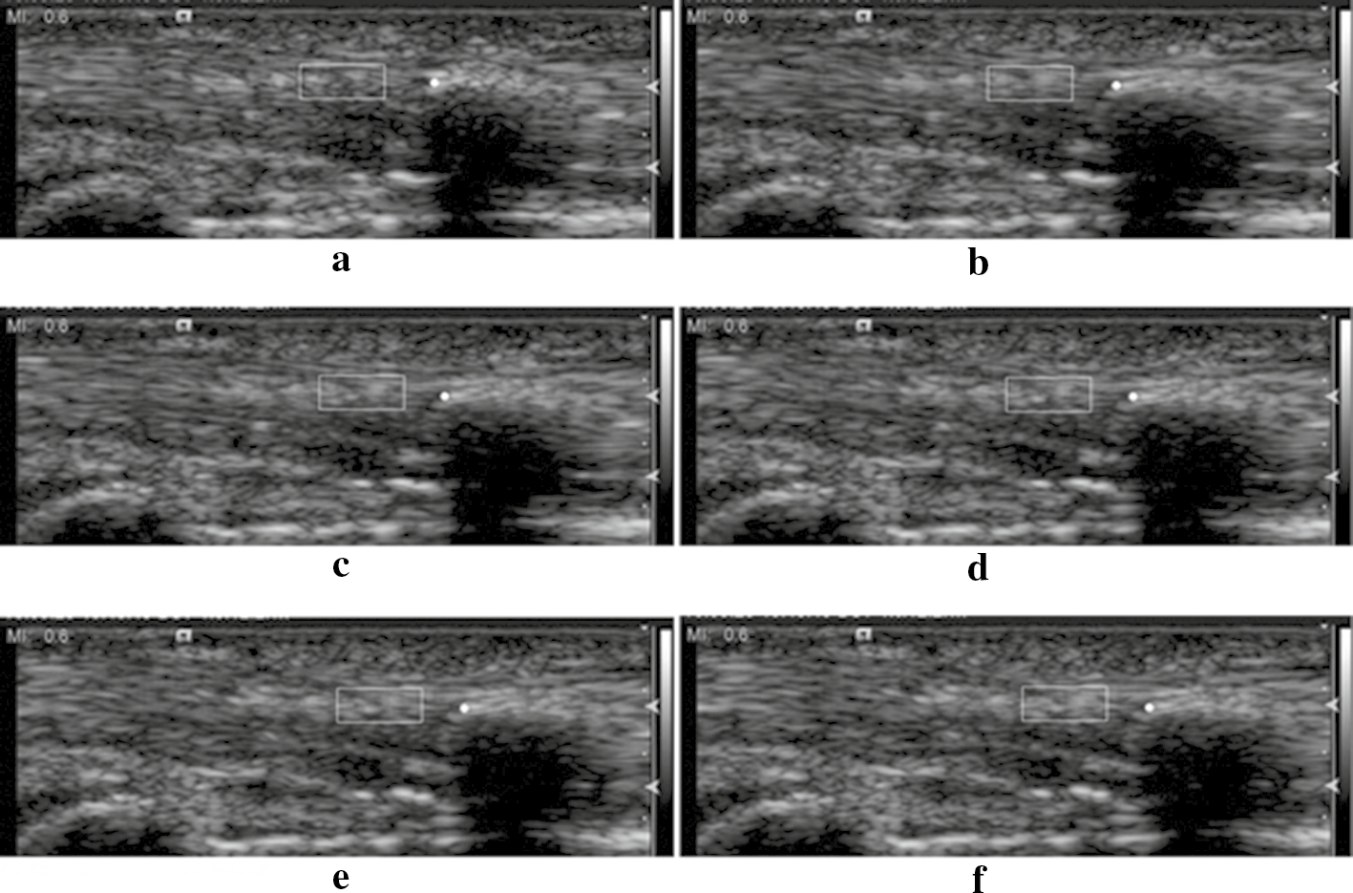
Tracking results and ground truths in cadaver data. **a** Initial frame, **b** frame 60, **c** frame 70, **d** frame 83, **e** frame 95, **f** frame 105

To validate the tracking results of tendon motions, the motions of the marker inside the FDP tendon of a cadaver were treated as the ground truth motions. The position of the marker was easily tracked manually because of its steady appearance during tendon sliding. The tracking region and the marker are in the same tendon; thus the distance between the tracking region and marker should be stable during the tracking. The distance between the tracking targets (white blocks) and the ground truths (white circles) were stable in all frames (Fig. 15a–f). Furthermore, Fig. 15b–f are the selected frames with λ = 10. The textures inside the tracking regions revealed large differences between Fig. 15a and f. However, the appearances on the sequentially selected regions from Fig. 15a–f progressively changed. This evidence shows that the tracking results matched the tendon motions throughout the tracking.

To quantify the tracking difference between the ground truth and the proposed method, two metrics were used in this experiment. The first metric, average absolute error (*E*_*a*_), compares the average instantaneous displacement between the proposed algorithm. The ground truth can be computed using the following equation:

9 $$E_{a} = \sum\limits_{t = 1}^{N} {\frac{{\left| {d_{GT,t} - d_{PA,t} } \right|}}{N}} ,$$

where *d*_*GT,t*_ and *d*_*PA,t*_ are the instantaneous displacements in the *t*th frame given by the ground truth and the proposed method, respectively, and *N* is the total number of frames.

Because the direction of tendon motion in our cadaver experiment was consistent, the second metric, relative error (*E*_*r*_), compares the total displacement of the proposed algorithm to the ground truth. *E*_*r*_ was computed using the following equation:

10 $$E_{r} = \left| {\frac{{D_{GT} - D_{PA} }}{{D_{GT} }}} \right|\times100\% ,$$

where *D*_*GT*_ and *D*_*PA*_ are the total displacements given by the ground truth and the proposed method, respectively. The total displacement stands for the displacement between the first frame and the final frame. The direction of tendon motion was mostly lateral; thus, only the X direction of displacement was considered for validation in our experiments. Optical flow and MKBM methods were also applied to compare the quantitative results. Furthermore, the adaptive MKBM, which is the original MKBM associated with linear interpolation, was also used. The two metrics were calculated for each motion case (Table 1). The results in five cadaver image sequences indicate the high accuracy of our proposed OFTB-MKBM algorithm than the other methods. Comparing the results of OFTB-MKBM and adaptive MKBM with MKBM, the two methods have better tracking results than MKBM since the tracking frames are not adjacent, which can prevent the speckle noise issue when the small target motion.

**Table 1:** The error metrics of the proposed results for cadaver (1 pixel = 0.0265 mm)

		1	2	3	4	5
Optical flow	E_r_ (%)	1.13	3.18	2.37	12.13	1.42
	E_a_ (pixel)	1.11	3.20	1.24	5.81	0.83
MKBM	E_r_ (%)	1.04	2.81	2.22	3.90	1.02
	E_a_ (pixel)	0.96	2.68	1.05	2.36	1.31
Adaptive MKBM	E_r_ (%)	0.65	2.52	2.08	3.33	0.96
	E_a_ (pixel)	0.49	2.51	0.61	1.81	1.02
OFTB-MKBM	E_r_ (%)	0.65	2.52	2.08	3.33	0.96
	E_a_ (pixel)	0.38	2.26	0.38	1.51	0.75

### OFTB-MKBM results of in vivo data

**Fig. 16:**
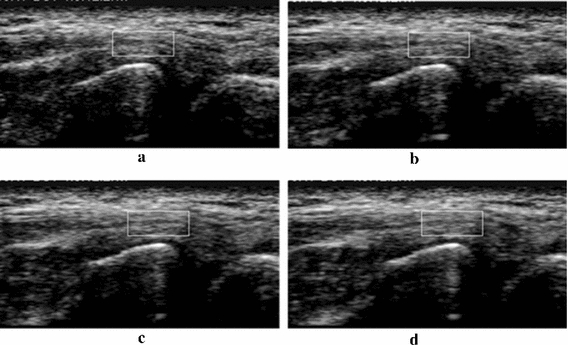
Tracking results for elbow. **a** Initial frame, **b** frame 87, **c** frame 118, **d** frame 140

**Fig. 17:**
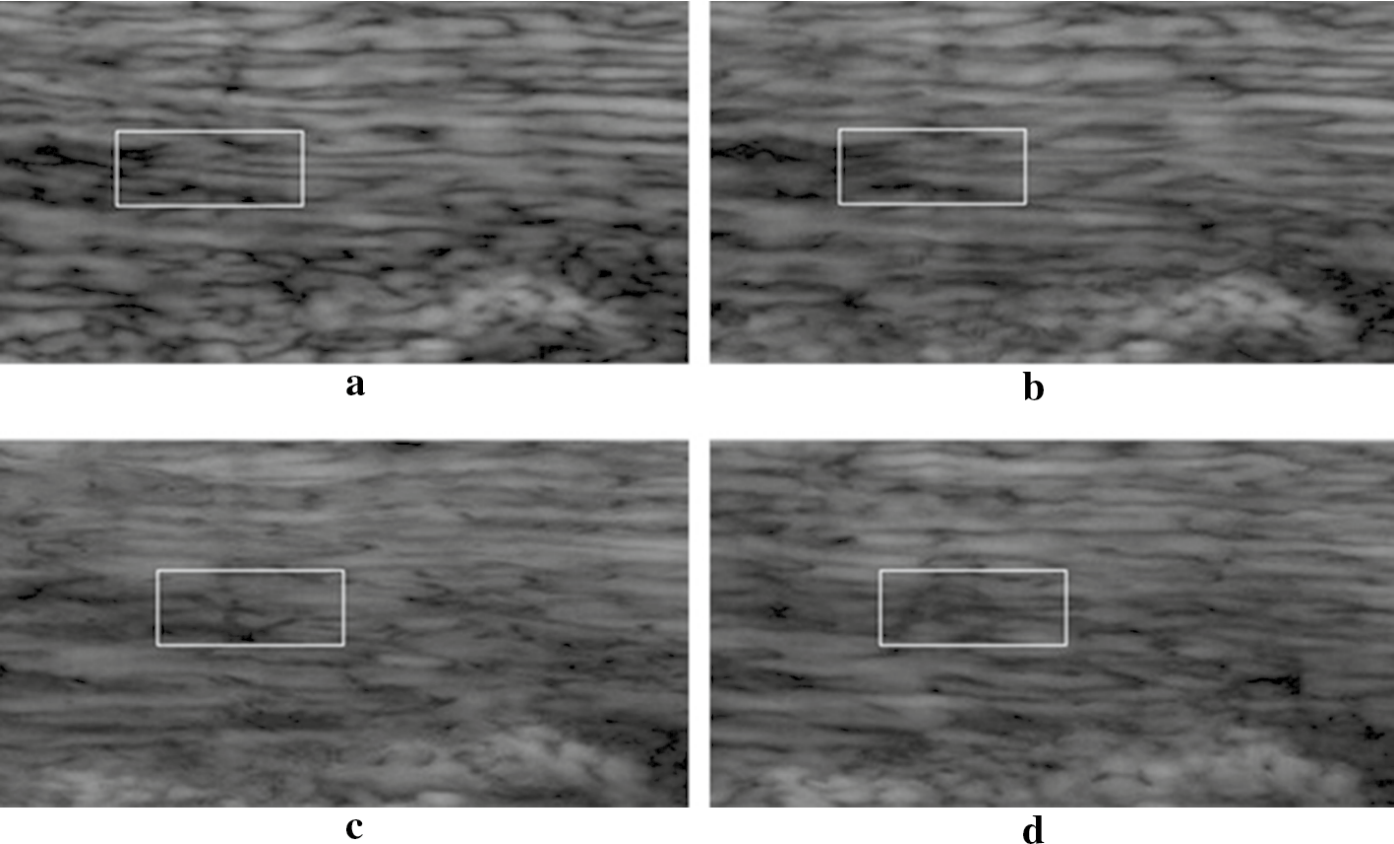
Tracking results for finger. **a** Initial frame, **b** frame 25, **c** frame 29, **d** frame 34

Figures 16 and 17 shows the tracking results of the proposed OFTB-MKBM for the elbow and finger. Figure 16a is the tracking target of the common extensor tendon for the first frame, and Fig. 17a is the tracking target of the FDP tendon for the first frame. Figure 16b–d are the tracking results at frames 87, 118, and 140, respectively, for elbow motion, and Fig. 17b–d are the tracking results at frames 25, 29, and 34, respectively, for finger motion. In both elbow and finger cases, the texture of tracking regions were similar and progressively changed. Moreover, based on the selected frame numbers of these two videos, our proposed method, which used λ = 10, can track the target with both fast and slow motion within an applicable displacement range.

### Comparisons with other methods

**Fig. 18:**
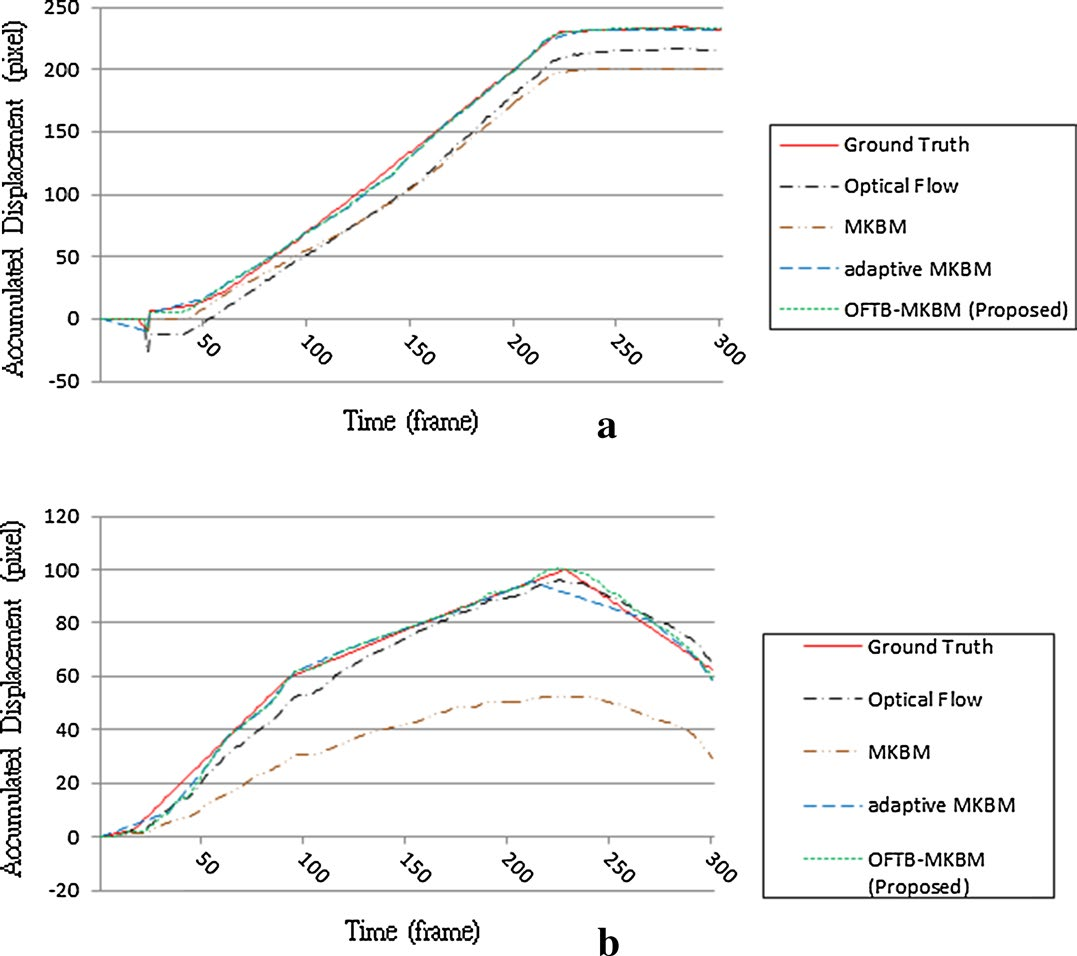
The tracking results of the four methods and the ground truth in the elbow. **a**, **b** Two different motion cases

**Fig. 19:**
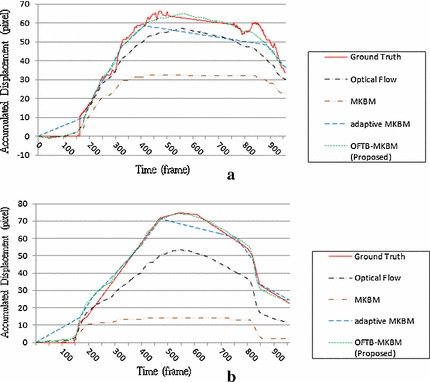
The tracking results of the four methods and the ground truth in the finger. **a**, **b** Two different motion cases

Figures 18 and 19 depict the ground truths and the tracking results for optical flow, MKBM, adaptive MKBM, and the proposed OFTB-MKBM in both the elbow and finger cases. Neither the optical flow method nor the MKBM tracked the tendon well for the entire motion sequence. Adaptive MKBM tracked tendon motion and yielded results similar to those of the ground truth (Fig. 18a, b); however, it yielded large deviations from the ground truth when the motion directions changed (Fig. 19a, b). The results of the proposed method (Additional files 6, 7, 8 and 9) are much closer to the ground truth than are those of the other methods.

To quantify the tracking errors for the various methods, the error metric (*E*_*a*_), computed using Eq. (9) was used. Table 2 shows the validation metrics of the four methods compared to the ground truth. The proposed OFTB-MKBM had better error metrics in all cases. The adaptive MKBM had error metrics close to those of the proposed method in six cases. However, the detailed displacement computed using linear interpolation created significant tracking errors in the remaining ten cases. The MKBM and optical flow methods had the most errors because of the accumulation of tracking errors or motion underestimation.

**Table 2:** Error metrics of the four methods compared with the ground truth

Case	Average absolute error E_a_ (pixels)							
	Elbow (1 pixel = 0.075 mm)				Finger (1 pixel = 0.0265 mm)			
	Optical flow	MKBM	Adaptive MKBM	OFTB-MKBM	Optical flow	MKBM	Adaptive MKBM	OFTB-MKBM
1	12.69	32.57	3.56	1.66	18.51	21.21	2.07	1.71
2	6.26	19.42	5.06	1.74	4.27	29.86	1.90	1.84
3	8.62	30.41	2.22	1.02	3.77	18.46	3.58	2.48
4	2.22	7.56	1.14	0.72	18.23	31.85	4.57	3.37
5	6.86	20.42	3.45	3.22	42.23	28.09	5.79	4.86
6	7.95	21.39	6.27	2.36	6.25	17.99	8.27	6.11
7	9.35	5.07	5.62	2.93	9.59	17.15	1.74	1.65
8	10.97	27.71	4.37	2.12	9.95	12.96	5.18	3.94

## Conclusion

We proposed a new ultrasound image tendon-tracking algorithm (Additional file 10). The OFTB-MKBM and MKBM methods were used to track the tendon motions in an elbow and in a finger. The accuracy of the proposed method was validated. Moreover, our proposed method yielded better tracking results than did the traditional optical flow and MKBM methods. The results interpolated based on optical flow were also better than were those of the adaptive MKBM method.

## Additional files

**Additional file 1.** Raw data (video).

**Additional file 2.** Raw data (video).

**Additional file 3.** Ground truth (point coordinates).

**Additional file 4.** Ground truth (point coordinates).

**Additional file 5.** Raw data (video).

**Additional file 6.** Tracking results (point coordinates).

**Additional file 7.** Tracking results (point coordinates).

**Additional file 8.** Tracking results (video).

**Additional file 9.** Tracking results (video).

**Additional file 10.** Source code of the software.

## Abbreviations

A1 pulley: first annular pulley
FDS: flexor digitorum superficialis
FDP: flexor digitorum profundus
OFTB-MKBM: optical-flow-trend-based multi-kernel block matching
MKBM: multi-kernel block matching
SAD: sum of absolute difference
